# Cochlear Implantation in Postlingually Deaf Adults is Time-sensitive Towards Positive Outcome: Prediction using Advanced Machine Learning Techniques

**DOI:** 10.1038/s41598-018-36404-1

**Published:** 2018-12-20

**Authors:** Hosung Kim, Woo Seok Kang, Hong Ju Park, Jee Yeon Lee, Jun Woo Park, Yehree Kim, Ji Won Seo, Min Young Kwak, Byung Chul Kang, Chan Joo Yang, Ben A. Duffy, Young Sang Cho, Sang-Youp Lee, Myung Whan Suh, Il Joon Moon, Joong Ho Ahn, Yang-Sun Cho, Seung Ha Oh, Jong Woo Chung

**Affiliations:** 1 Department of Neurology, USC Stevens Neuroimaging and Informatics Institute, Keck School of Medicine, University of Southern California, Los Angeles, USA; 2 Department of Otolaryngology, Asan Medical Center, University of Ulsan College of Medicine, Seoul, South Korea; 3 Department of Otorhinolaryngology-Head and Neck Surgery, Ulsan University Hospital, University of Ulsan College of Medicine, Ulsan, Korea; 4 Department of Otolaryngology, Hanil General Hospital, Seoul, South Korea; 5 Department of Otorhinolaryngology-Head and Neck Surgery, Samsung Medical Center, Sungkyunkwan University School of Medicine, Seoul, South Korea; 6 Department of Otorhinolaryngology-Head and Neck Surgery, Seoul National University Hospital, Seoul National University College of Medicine, Seoul, South Korea

## Abstract

Given our aging society and the prevalence of age-related hearing loss that often develops during adulthood, hearing loss is a common public health issue affecting almost all older adults. Moderate-to-moderately severe hearing loss can usually be corrected with hearing aids; however, severe-to-profound hearing loss often requires a cochlear implant (CI). However, post-operative CI results vary, and the performance of the previous prediction models is limited, indicating that a new approach is needed. For postlingually deaf adults (n de120) who received CI with full insertion, we predicted CI outcomes using a Random-Forest Regression (RFR) model and investigated the effect of preoperative factors on CI outcomes. Postoperative word recognition scores (WRS) served as the dependent variable to predict. Predictors included duration of deafness (DoD), age at CI operation (ageCI), duration of hearing-aid use (DoHA), preoperative hearing threshold and sentence recognition score. Prediction accuracy was evaluated using mean absolute error (MAE) and Pearson’s correlation coefficient *r* between the true WRS and predicted WRS. The fitting using a linear model resulted in prediction of WRS with *r* = 0.7 and MAE = 15.6 ± 9. RFR outperformed the linear model (*r* = 0.96, MAE = 6.1 ± 4.7, p < 0.00001). Cross-hospital data validation showed reliable performance using RFR (*r* = 0.91, MAE = 9.6 ± 5.2). The contribution of DoD to prediction was the highest (MAE increase when omitted: 14.8), followed by ageCI (8.9) and DoHA (7.5). After CI, patients with DoD < 10 years presented better WRSs and smaller variations (p < 0.01) than those with longer DoD. Better WRS was also explained by younger age at CI and longer-term DoHA. Machine learning demonstrated a robust prediction performance for CI outcomes in postlingually deaf adults across different institutes, providing a reference value for counseling patients considering CI. Health care providers should be aware that the patients with severe-to-profound hearing loss who cannot have benefit from hearing aids need to proceed with CI as soon as possible and should continue using hearing aids until after CI operation.

## Introduction

Given our aging society and the prevalence of age-related hearing loss that often develops during adulthood, hearing loss is a common public health issue affecting almost all older adults. Cochlear implants (CI) are most commonly used to treat adults as well as prelingual deaf children with severe to profound hearing loss who cannot benefit from hearing aids^1–3^. As of December 2012, approximately 324,200 registered CI devices have been implanted worldwide. In the United States, roughly 58,000 devices have been implanted in adults and 38,000 in children (https://www.nidcd.nih.gov/health/cochlear-implants).

Performance of CI in adults depends on several preoperative factors. Many factors including age at CI operation, duration of hearing loss, the presence of residual hearing, previous hearing aid use, and the presence of cochlear anomaly are considered to be related to the outcomes. Other factors including the technique of CI operation, etiology and the brand of device also have an effect on CI performance^4–7^. Some studies reported a negative relationship between duration of deafness (DoD) and postoperative speech and its greater role when combined with residual hearing^5,6,8–10^, whereas others reported that DoD might have no or even a positive relationship with speech recognition^4,7^. Moreover, postoperative speech recognition performance in younger adults has been observed to be better than in older adult CI users^4–7,9–12^, though others have reported no differences between middle-aged and elderly patients (over 70 years of age) except in the hearing ability in noise^13,14^. Though the majority of postlingually deaf adults restore meaningful speech recognition with CI, the large variation of outcome across individuals makes predictions using conventional statistical methods suboptimal^4–6,8,15–17^.

To explain better a large variation in outcomes, it is necessary to perform a multivariate analysis of all predictive factors as well as possible nonlinearities. A reliable prediction model for postCI outcome is needed to improve preoperative counseling and potentially benefit the deaf patients in clinical practice. We thus proposed to build predictive models of postCI outcomes by combining common preoperative variables with multivariate regression modeling using a nonlinear machine learning approach as well as a general linear model. Such machine-learning approaches have been successfully used to improve disease diagnosis or predictions across various conditions^18–20^. We also validated the fitted model using data from two other independent hospitals and investigated the effect of preoperative factors on CI outcomes.

## Results

The study cohort consisted of 50 men and 70 women. The mean age at CI operation was 51.2 ± 13.2 years (range, 21.0–80.3 years, Table 1). The mean device length used at the time of the latest language assessment was 56.7 ± 33.4 months (range, 24–168 months). Most patients (98/120) were implanted with CI devices from Cochlear Corp. (Lane Cove, New South Wales, Australia) with various types of electrodes and speech processors. Twenty were with Devices from MED-EL (Innsbruck, Austria) and 2 with Advanced Bionics, Corp. (Sylmar, CA, USA). Sixty-eight patients were implanted in the right ear and 52 in the left ear. One patient had bilateral CIs, and only data from the first implanted ear were used in this analysis.

**Table 1:** Demographic and audiologic results in postlingually deaf adults with CI (N = 121).

Variables	Mean	SD	Range
Age at CI operation, AgeCI (yr)	51.2 yr	13.2	21.0–80.3 yr
Duration of deafness, DoD (yr)	13.8 yr	13.2	0.1–50 yr
Duration of hearing aid use, DoHA (yr)	5.2 yr	8.0	0–46 yr
Postoperative follow-up duration (Mo)	56.7 months	33.4	24–168 months
**Preoperative audiologic results**			
Preoperative PTA in CI ear (dB HL)	103.5 dB HL	13.9	66–120 dB HL
Preoperative PTA in the contralateral ear (dB HL)	98.4 dB HL	15.2	70–120 dB HL
Preoperative best-aided WRS in CI ear (%)	4.5%	9.0	0–48%
Preoperative best-aided WRS in the contralateral ear (%)	10.2%	15.0	0–60%
Preoperative best-aided sentence recognition score (%)	9.7%	15.9	0–48%
**Postoperative audiologic results**			
Postoperative CI-aided SRT (dB HL)	26.4 dB HL	5.5	16–48 dB HL
Postoperative CI-aided PTA (dB HL)	30.3 dB HL	5.9	19–45 dB HL
Postoperative CI-aided WRS (%)	67.0%	21.6	4–100%
Postoperative CI-aided sentence recognition score (%)	95.1%	14.4	18–100%

CI = cochlear implant; PTA = pure-tone averages; WRS = word recognition score; SRT = speech recognition threshold.

The mean preoperative pure-tone average (PTA) was 103.5 ± 13.9 dB HL (range, 66–120 dB HL) in the ear with CI and 98.4 ± 15.2 dB HL (range, 70–120 dB HL) in the contralateral ear. The mean preoperative best-aided word recognition score (WRS) was 4.5 ± 9.0% (range, 0–48%) in the ear with CI and 10.2 ± 15.0% (range, 0–60%) in the contralateral ear. The mean preoperative best-aided sentence recognition score was 9.7 ± 15.9% (range, 0–48%).

**Figure 1:**
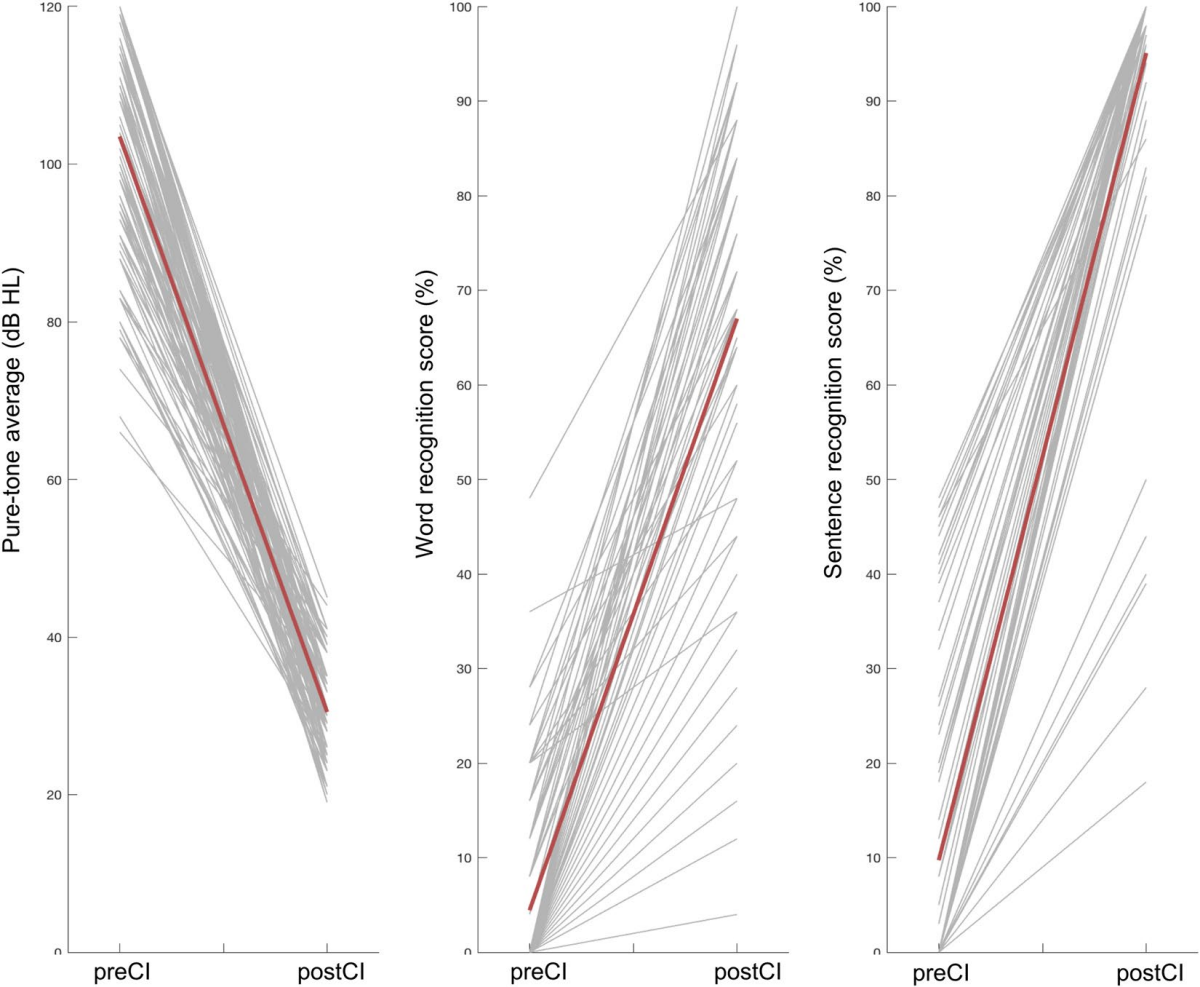
Changes in audiologic test results before and after the CI operation. Postoperative follow-up in each individual was made for 2 years. Shown are decrease in postCI hearing thresholds (pure-tone averages) and increase in word and sentence recognition scores. Grey lines represent individual patients and red their mean changes.

Postoperatively, the mean postoperative sound-field PTAs was 30.3 ± 5.9 dB HL (19–45 dB HL) with significant improvement with their CIs compared with their preoperative PTAs (Fig. 1). The mean postoperative CI-aided WRS was 67.0 ± 21.6% (range, 0–100%) and the mean postoperative CI-aided sentence recognition score was 95.1 ± 14.4% (range, 18–100%) with significant improvements.

### General linear models

**Figure 2:**
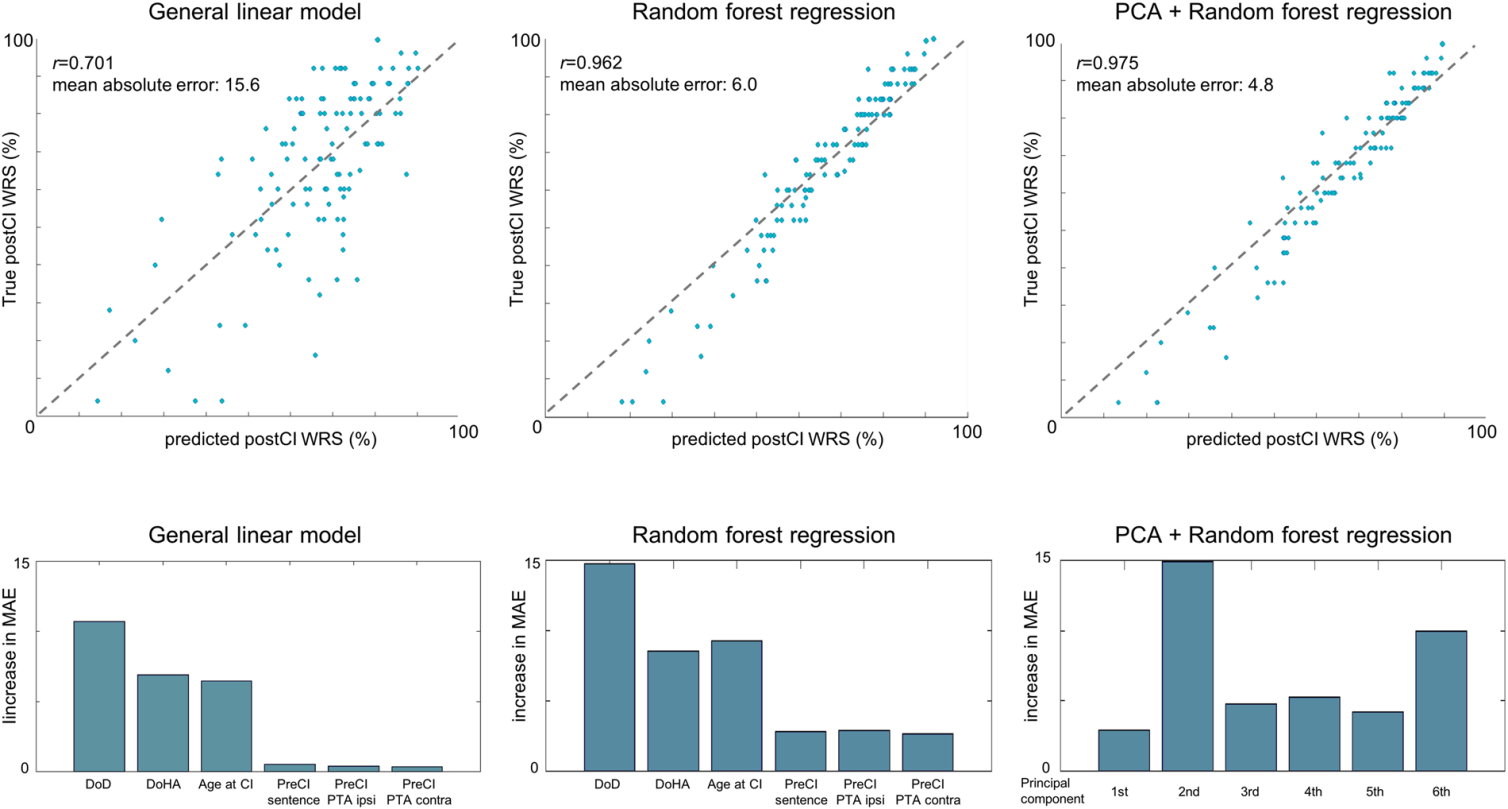
Predictive performance of postoperative word recognition using different models including a general linear model (GLM; 1st column) and a random forest regression (RFR; 2nd column). We also performed principal component analysis (PCA) to reconstruct features regarding covariance of the original predictive variables and fed the new features to the RFR (3^rd^ column). Upper: prediction results – blue circles indicate individual patients. Gray dot lines represent the ideal fitting where the error is 0. The farther a circle is from the line, the less accurate its prediction is. The nonlinear RFR outperformed the result of GLM. The PCA + RFR model further improved slightly the result of RFR only. Lower: Importance of each feature in terms of decrease in mean absolute error (MAE) when the given feature was omitted from the prediction process. Abbreviations: DoD – duration of deafness, DoHA – duration of hearing aid use, Age at CI – age at cochlear implantation, PreCI Sentence - sentence recognition score measured preoperatively; preCI PTA ipsi/contra – preoperative PTA in CI ear/in the contralateral ear; WRS: word recognition score.

**Figure 3:**
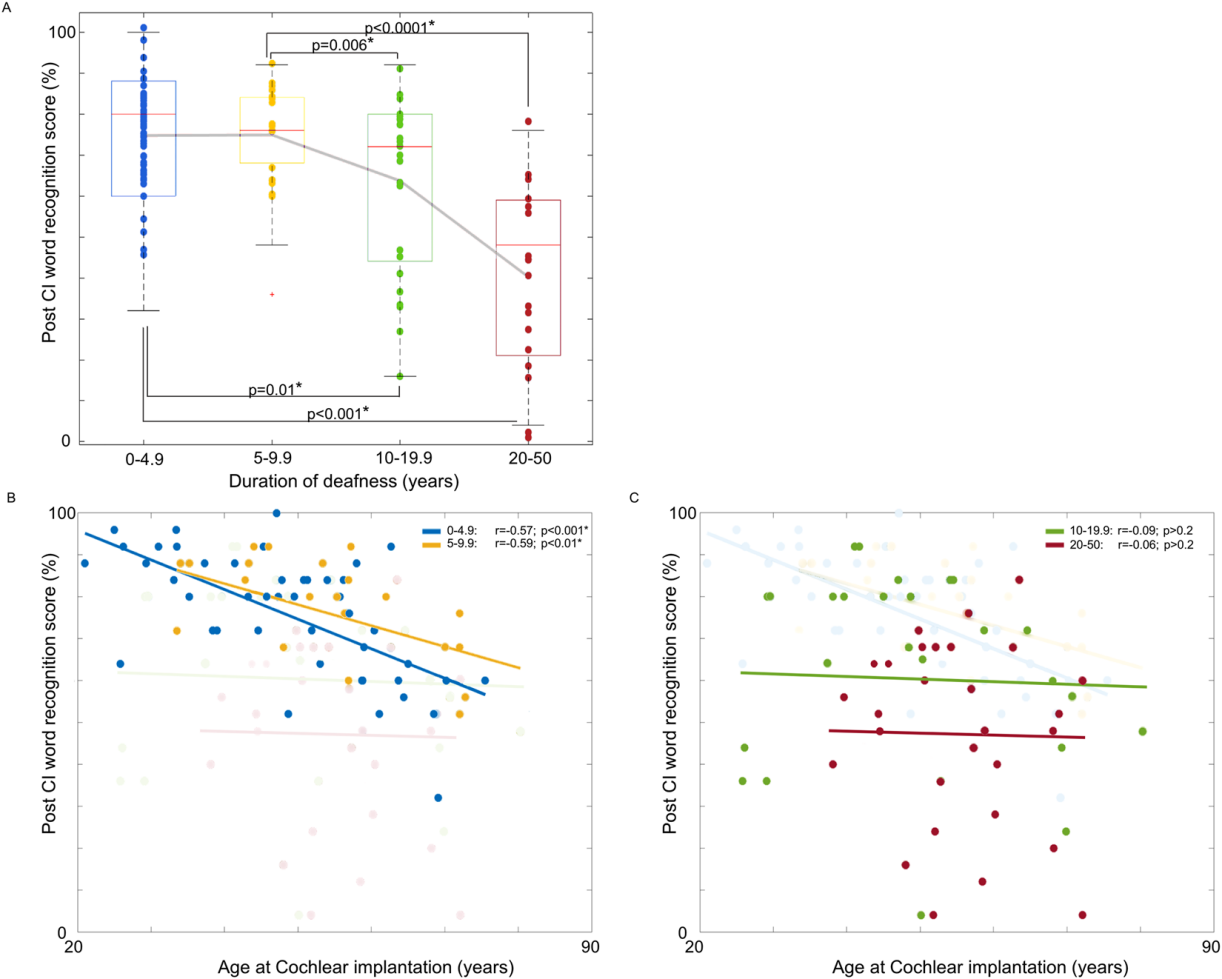
Association of postCI outcomes with DoD and age at CI operation. (**A**) When DoD was longer than 10 years, postoperative WRS was significantly lower compared to when DoD was shorter than 10 years. The difference in postCI WRS became even larger when comparing patients with DoD of 20 years or longer to those with DoD of shorter than 10 years. (**B**) PostCI WRS significantly correlated with age at CI operation in subgroups of patients with DoD of 0–4.9 years and 5–9.9 years. (**C**) No such correlation was found in patients with DoD of 10–19 years, and those with 20 years or longer as much larger variability across individuals were observed in these groups. In (**B**), and (**C**), transparent data points and lines were used to help the comparison between the four subgroups.

The fitting of general linear models (GLM)s (Fig. 2) resulted in prediction performance with correlation coefficient *r* = 0.7 and mean absolute error (MAE) of 15.6 ± 9.5 (mean ± standard deviation). Computation of the feature importance showed that the contribution of duration of deafness (DoD) to the prediction was the largest (MAE increase when omitted: 10.7), followed by duration of hearing aid use (DoHA; 6.8), and age at CI operation (AgeCI; 6.6). The contributions of PreCI sentence recognition score (0.5), and preCI hearing threshold (ipsilateral: 0.4, contralateral: 0.3) were much smaller. Post-hoc analyses indeed showed that AgeCI, DoD, and DoHA were good predictors as they significantly correlated with postCI WRS (AgeCI: r = −0.33, p < 0.0001; DoD: r = −0.61, p < 0.00001; DoHA: r = 0.44, p < 0.00001). To better understand whether DoD, ageCI or their combination lead to more positive post-surgical outcomes, we assessed the association of ageCI and DoD with postCI outcome. We found that the postCI WRS was different across the four DoD groups (ANOVA; F = 59, p < 0.0001, Fig. 3). The postCI WRS in the subgroups of patients with DoD of 0–4.9 years (postCI WRS: 75 ± 19%) or 5–9.9 years (75 ± 11%) was significantly higher compared to those with DoD of 10–19.9 years (59 ± 21%) or 20 years or longer (40 ± 22%) (t > 2.5, p < 0.01). Furthermore, ageCI was significantly associated with the postCI WRS in the groups with DoD of 0–4.9 years and 5–9.9 years (r < −0.57; p < 0.01) whereas such a relationship was not found in the group with DoD of 10–19.9 years and 20 years or longer (−0.05 > r > −0.1; p > 0.2).

### Machine learning prediction model

**Figure 4:**
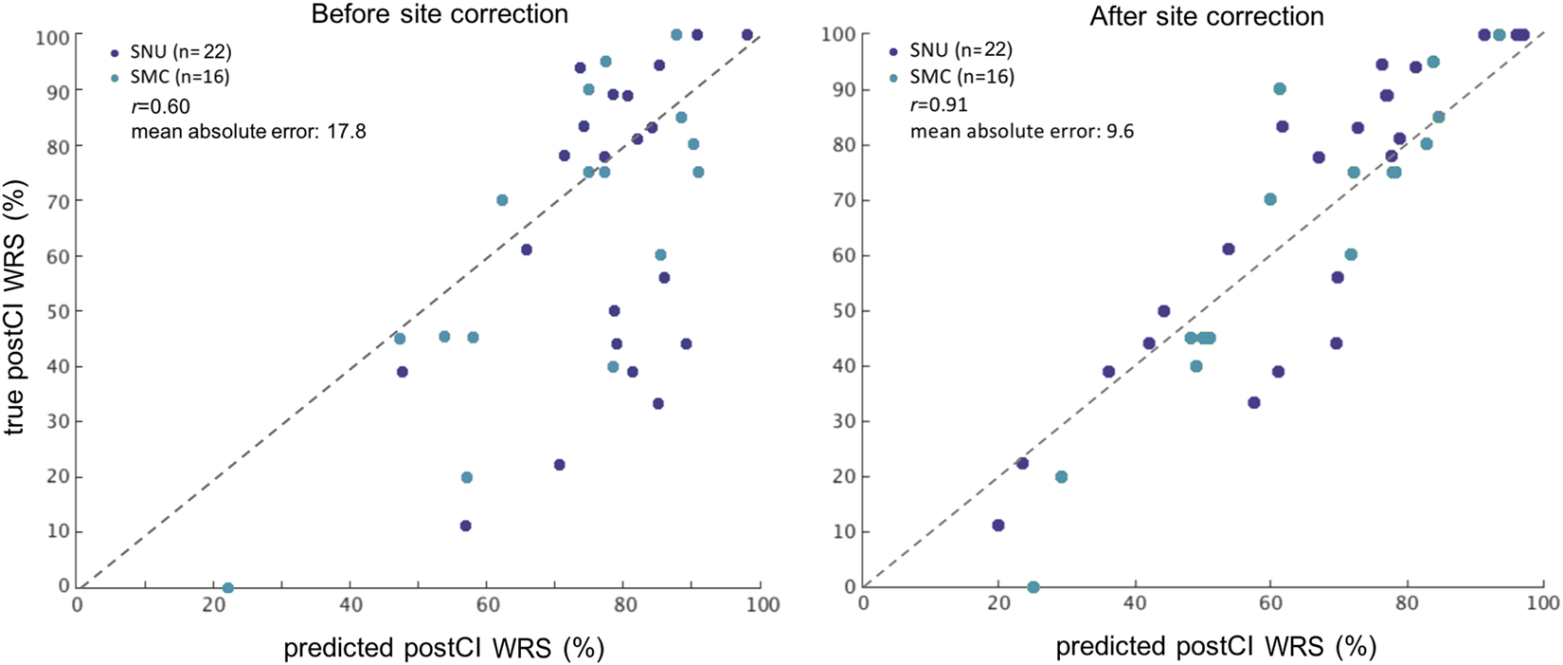
Cross-validation of the trained random forest regression on the mixed cohorts of data from other institutes (SMC and SNU). The prediction results on these cohorts using the random forest regression model which had been trained using the main data from Asan Medical Center (AMC) are shown. Results before considering the site bias (**A**), results after correcting the site bias (**B**). The bias was assumed to be linear and thus corrected using the linear model which was performed using a leave-one-out approach (per site) to determine the coefficient of the slop for the test patient. The inverse transformation was applied to the determined coefficient to obtain the new result in the right panel.

The random forest regression (RFR) machine learning yielded superior prediction performance to the GLM with r = 0.962 and MAE of 6.0 ± 4.7 (t = 9.9; p < 0.00001, Fig. 2). Computation of the feature importance showed that DoD contributed most largely to the prediction (MAE increase when omitted: 14.8), followed by AgeCI (8.9), DoHA (7.5), preCI hearing threshold (ipsilateral: 3.7, contralateral: 2.9) and PreCI sentence recognition score (3.2). Feeding only the first three most important features (i.e., DoD, AgeCI, DoHA) into the RFR resulted in a similarly high accuracy of prediction (r = 0.931; MAE = 7.1 ± 5.5; vs. GLM: t = 9.5; p < 0.00001). The combination of PCA and the RFR showed the best performance with r = 0.975 and MAE of 4.8 ± 4.4 (vs. GML: t = 11.4; p < 0.00001). Finally, cross-validation of the trained RFR model on the mixed cohort of Seoul National University hospital (SNU; n = 22) and Samsung Medical Center (SMC; n = 16) data showed a significantly higher MAE (17.1 vs. 6.0, Fig. 4), likely due to the site bias related to the difference in the test materials used for measuring the WRS (different words and different numbers of words: Asan Medical Center [AMC] = 25; SNU = 18; SMC = 20). Assuming this bias to be linear, we applied a post-hoc correction using a GLM which included the site as a covariate when pooling all the three sites data in the fitting. After correction, RFR on the fitted data, resulted in a significantly reduced MAE for the test cohort (9.6 ± 5.2).

To address the site bias without the post-hoc process, we included the site information as a variable in the RFR model and train and test it with the entire set of the three site data using a leave-one-out cross-validation. To avoid overfitting, we used an ensemble learning approach using the LS-boosting with a learning rate of 0.05 and the number of learning cycles of 100^21^. The inclusion of the site variable in the RFR and performing the ensemble method resulted in the mean MAE of 9.7 and r = 0.90 across the three site datasets (Supplementary Figure 1), which was similar to the result when the post-hoc correction was used.

## Discussion

We adopted a machine learning modeling using easy-to-acquire clinical data (e.g. DoD, age at CI, DoHA, preoperative PTA and sentence recognition score) to predict postoperative WRS in postlingually deaf adult CI users. Our advanced nonlinear regression combined with PCA best predicted the outcome with a high accuracy of 95.2%. This performance is superior to previously reported linear predictive models^4–6,8,10,17^. One interesting finding was that the precision of prediction using the three most important preoperative factors only (i.e., DoD, Age at CI, and DoHA) could result in a comparably high accuracy (93.7%). Other factors, preoperative residual hearing (preCI hearing threshold and sentence recognition score), were also positively associated with CI outcomes, though they contributed little to prediction of postCI WRS^4,5,8,10,12,17^. Our validation across three different hospitals suggested that the regression model is yet required to consider possible site bias prior to the testing in order to achieve accurate prediction across different sites. Possible reasons for the bias could related to differences in the test materials and conditions used in each clinic.

In this study, in line with previous reports^4,8,10,15,16^, DoD was the most important predictor of CI outcomes. The gradual decrease in spiral ganglion cell population by age may get worse due to a longer duration of deafness and a late operation of CI, leaving fewer spiral ganglion neurons available for stimulation by CIs^22–24^. Moreover, aging with a late CI operation can decline top-down cognitive processing required for auditory function and decoding of the input provided by the CI, thus negatively influencing CI outcomes^12,25,26^. While the negative relationship between age at CI and outcome was hypothesized, our data showed that this was significant only when DoD was less than 10 years. On the other hand, a larger individual variation in postCI WRS was observed when DoD was ≥10 years, suggesting additional factors influenced outcome.

A possible mechanism explaining such a large individual variation of the CI outcome is cross-modal plasticity. Cross-modal functional re-organization of visual, somatosensory and auditory cortices can occur as a result of decreased or abnormal sensory input, whereby the cortical region of the deprived modality becomes vulnerable to the recruitment by the remaining other intact sensory modalities^27–29^. This neural activity was detrimental to auditory performance in CI users, especially after a long duration of auditory deprivation. This can begin in the early stages of hearing loss and may persist even when hearing is restored by CI^5,28,30–33^.

In accordance with the reports showing the presence of the reversibility of cortical resource allocation^27,34–37^, our findings suggest that central re-organization is mostly reversible when deafness lasts less than 10 years. However, postCI WRS declined significantly for patients with 10 years or longer DoD and aggravated further for those with 20 years or longer DoD (Fig. 2C). Therefore, these together suggest that the reversibility of central re-organization becomes partial in some patients after 10 years of deafness, implicating that this might be a sensitive period for postlingually deaf adults to obtain good postCI outcomes.

**Figure 5:**
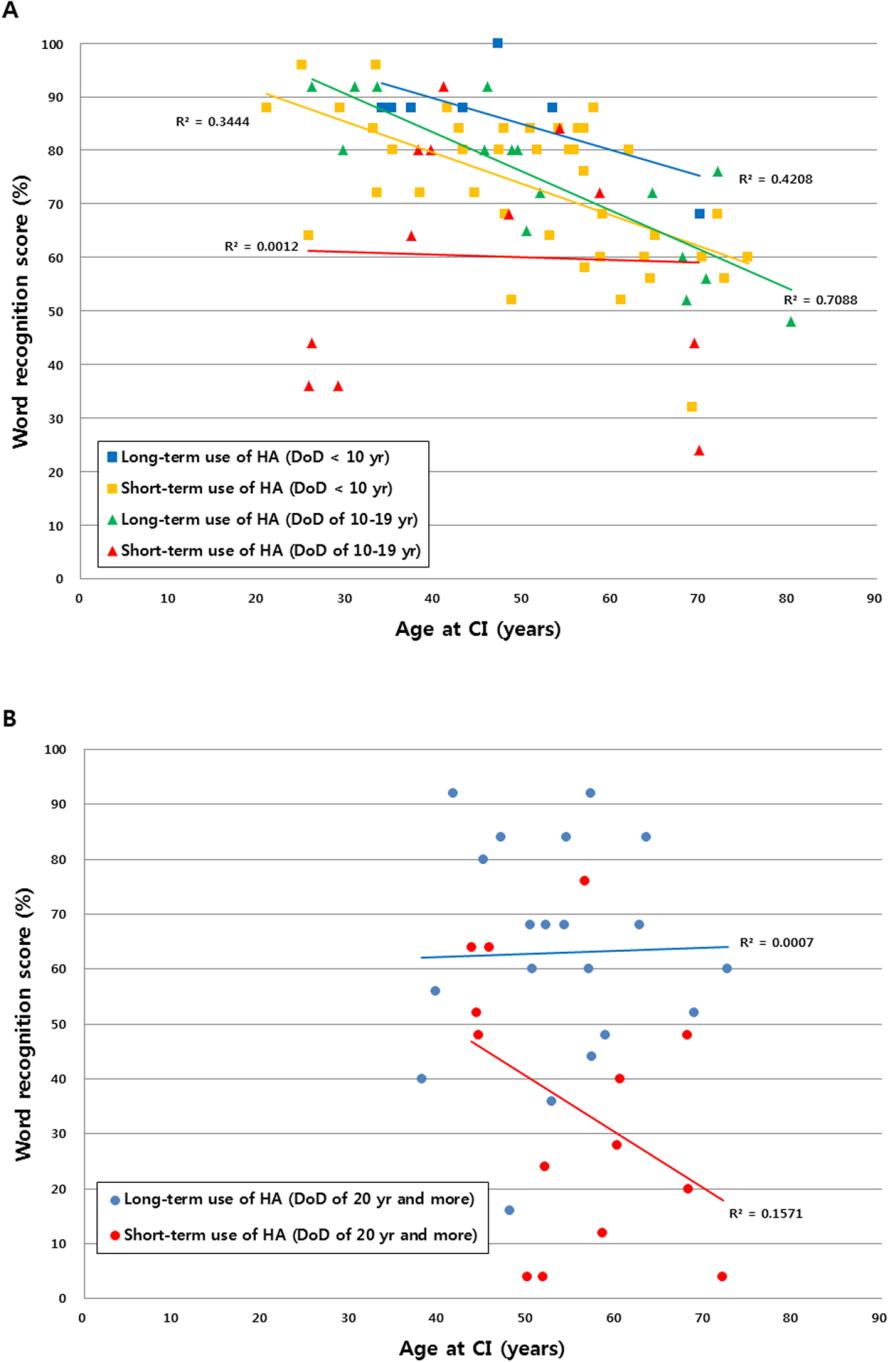
Linear relationship of word recognition scores (WRS) with age at CI operation for the subgroups of our patients based whether short (<10 years) or intermediate (10–19 years) or long (>20 years) duration of deafness (DoD) and whether short-term (<2 years) or long-term (>5 years) preoperative use of hearing aids. (**A**) Patients with short DoD (regardless of short-term or long-term hearing aid use) and those with intermediate DoD and long-term hearing aid use showed significant correlations of age at CI with postCI WRS operation. On the other hand, patients with intermediate DoD with poor hearing aid use show no such a correlation (r = 0.3; p = 0.4), suggesting that age at CI operation is not an important outcome predictor in this subgroup. (**B**) Patients with long DoD regardless of short-term or long-term hearing aid use showed no correlation of age at CI operation with postCI WRS (r < 0.3; p > 0.3). Patients with long DoD and short-term hearing aid use displayed the poorest postCI WRS (mean = 32%).

Auditory input from hearing rehabilitation to the better hearing ear may maintain the ability of the central auditory pathway to decode speech information and may further slowdown or restore the related cross-modal reorganization by trophic effects on crossed pathways, regardless of the side of ear in postlingual deaf adults^5,8,10,37–39^. Indeed, duration of hearing aid use was the third most important predictor of CI outcomes. Constant use of hearing aids might maintain the functionality of the auditory system for the future CI. Among the patients with 20 years or longer DoD in our study, those with no or short-term (<2 year) hearing aid use before CI showed poorer postCI WRS than long-term hearing aid users (5 year+; Fig. 5). This finding suggests that the long-term use of hearing aids before CI additionally benefits the outcome of CI operation.

In congenitally prelingual deafness, the absence of sensory input until the age of seven affects normal development and connectivity of the auditory cortex, resulting in irreversible deficits in speech recognition and language learning^2,28,29,37,40–43^. Because the auditory system in postlingually deaf adults had been already established prior to the onset of deafness, 94% of adult CI users achieved good postCI sentence recognition scores larger than 80% and many (82%) showed WRS of 50% or more (Fig. 1). Though most patients showed relatively good WRS, some patients with DoD of 10 years or more resulted in relatively poor WRS (<50%), suggesting that a DoD of 10 years might be a sensitive period after which central re-allocations started to become irreversible in some patients (Fig. 3C). The sentence recognition test consisted of commonly used words and patients could estimate words by context. Due to this easy-to-achieve score, the sentence recognition score was not considered a good CI outcome measure and WRS served as the only outcome measurement in the current study (Fig. 1).

## Conclusions

Our machine learning model, which is currently prepared for the compilation of the code, open-source licensing and uploading the software to a public domain (https://github.com), demonstrated a robust prediction performance for CI outcomes in postlingually deaf adults across different institutes, providing a reference value for counseling patients considering CI. Health care providers should be aware that the patients with severe-to-profound hearing loss who cannot have benefit from hearing aids need to proceed with CI as soon as possible and should continue using hearing aids until after CI operation.

## Methods

Among 1,451 patients who underwent CI operation at Otology Clinic of Asan Medical Center from April 1999 to December 2016, 529 were adults. Among them, 402 were postlingual adults, and inner ear anatomy was normal in 275. This is a retrospective study using a cohort of postlingually deaf adults (n = 120), who underwent fully inserted CI surgery and were followed up for more than 2 years. Postlingual deafness was defined as a severe-to-profound HL that began after 10 years of age. For patients with bilateral CIs, only outcomes for the first implanted ear were analyzed. This study was approved by the institutional review boards of participating institutes. Approval of the institutional review board at the host institute (Asan Medical Center) included a ‘waiver of consent’ to allow sharing of data with collaborators without seeking further consent from participants because personal identifiers are not included in the data.

We used the following preoperative variables as predictors of postCI WRS: DoD, ageCI, DoHA, and PTAs of the ipsilateral and contralateral ears to the CI and preoperative sentence recognition score (Table 1). DoD was determined by a review of available medical records. The duration of deafness was determined as the duration during which the patient reported little or no hearing in both ears before the CI operation. Some patients tried to hear auditory input through hearing aids though the benefit might have been minimal. DoHA was defined as the duration of hearing aid use. As the definite causes of deafness in most of the patients was unknown, this factor was not included as a predictor in the analysis.

We used the scores of open-set monosyllabic word recognition test in quiet, which is used for conventional speech audiometry, as the outcome variable. Open-set tests were those in which no response alternatives were provided and the listener repeated what was heard; theoretically, there were an unlimited number of response possibilities. Only the most recent audiologic evaluation was included in the postCI analysis. Testing was conducted in a sound-treated booth. Score was measured via monitored live voice from a loudspeaker positioned at 0 degree azimuth approximately 1 m from the subject using 25 monosyllabic words. The presenting sound pressure level was at speech reception threshold +40 dB sound pressure level at the best-aided condition. PTAs were determined by averaging the pure-tone air-conduction thresholds measured at 500, 1,000, 2,000, and 4,000 Hz. When there was no response to a presented tone at the limits of the audiometer, a level of 120 dB was assigned. Postoperative testing was conducted using the CI alone without the use of a hearing aid in the contralateral ear.

To assess the association of the predictors with postCI WRS, we first used GLMs that addressed covariate effects of the predicting variables as independent variables and postCI WRS as the dependent variable. We also included sex in the GLM as a covariate. In a separate analysis, we performed nonlinear machine learning that fitted all variables to postCI WRS using the RFR. In contrast to typical linear algorithms, this nonlinear method allows a robust and highly reproducible prediction using feature weighting and bootstrapping^44^. The following parameters that yielded the best performance were set empirically: #trees = 50; #permutations = 1000; node size at the terminal ≥3. Predictive accuracy was evaluated using the MAE, and the Pearson’s correlation coefficient between the true WRS and predicted WRS for each of GLM and RFR approaches. To determine the importance of each predictor, we measured the increase in the MAE when a given variable was omitted in the regression model relative to when it was included. We used a leave-one-out cross-validation to avoid bias. The reproducibility of the trained model was assessed by testing the prediction in the mixed cohort from other institutes, Samsung Medical Center (SMC) and Seoul National University Hospital (SNU). Finally, as some of the predicting variables were seen to correlate each other (DoD and DoHA: r = 0.5; DoD and AgeCI: r = 0.17) and they were therefore not entirely independent, we performed the principal component analysis (PCA) to reconstruct the predicting features that were orthogonal each other. We repeated the regression process using the principal components as predictors.

## Electronic supplementary material

Suppl Figure 1
