# Supplementary material for: Cochlear Implantation in Postlingually Deaf Adults is Time-sensitive Towards Positive Outcome: Prediction using Advanced Machine Learning Techniques
Source: Sci Rep. 2018 Dec 20;8:18004. doi: 10.1038/s41598-018-36404-1 (PMC6301958; doi:10.1038/s41598-018-36404-1)
Supplement: Supplementary file 1 — Suppl Figure 1 [file 41598_2018_36404_MOESM1_ESM.docx]

**Cochlear Implantation in Postlingually Deaf Adults is Time-sensitive Towards Positive Outcome: Prediction using Advanced Machine Learning Techniques**

Hosung Kim^*,1^, Woo Seok Kang^*,2^, Hong Ju Park^2^, Jee Yeon Lee^2^, Jun Woo Park^2^, Yehree Kim^2^, Ji Won Seo^2^, Min Young Kwak^2^, Byung Chul Kang^3^, Chan Joo Yang^4^, Ben A Duffy^1^, Young Sang Cho^5^, Sang-Youp Lee^6^, Myung Whan Suh^6^, Il Joon Moon^5^, Joong Ho Ahn^2^, Yang-Sun Cho^5^, Seung Ha Oh^6^, Jong Woo Chung^2^

^1^Department of Neurology, USC Stevens Neuroimaging and Informatics Institute, Keck School of Medicine, University of Southern California;^2^Department of Otolaryngology, Asan Medical Center, University of Ulsan College of Medicine, Seoul, South Korea; ^3^Department of Otorhinolaryngology-Head and Neck Surgery, Ulsan University Hospital, University of Ulsan College of Medicine, Ulsan, Korea; ^4^Department of Otolaryngology, Hanil General Hospital, Seoul, South Korea; ^5^Department of Otorhinolaryngology-Head and Neck Surgery, Samsung Medical Center, Sungkyunkwan University School of Medicine; and ^6^Department of Otorhinolaryngology-Head and Neck Surgery, Seoul National University Hospital, Seoul National University College of Medicine

^*^Hosung Kim and Woo Seok Kang contributed equally to this work.

Corresponding author:

Hong Ju Park, MD, PhD

Department of Otorhinolaryngology-Head and Neck Surgery

Asan Medical Center, University of Ulsan College of Medicine

86 Asanbyeongwon-gil, Songpa-gu, Seoul 138-736, Korea

E-mail: dzness@amc.seoul.kr

Tel: +82-2-3010-3700

Fax: +82-2-489-2773

Competing interests: None

Sponsorships: None

Funding source: None


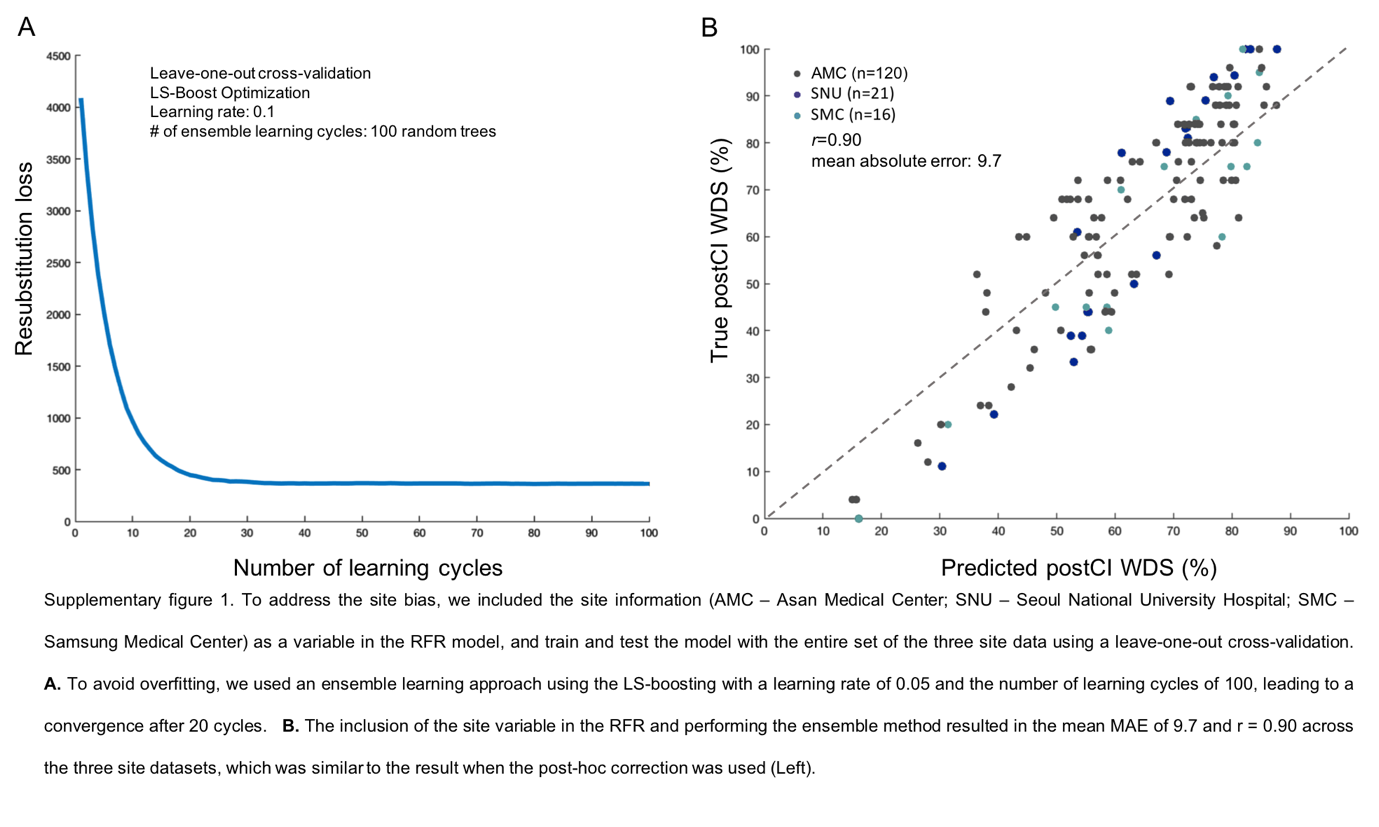


Supplementary Figure 1. To address the site bias, we included the site information (AMC – Asan Medical Center; SNU – Seoul National University Hospital; SMC – Samsung Medical Center) as a variable in the RFR model, and train and test the model with the entire set of the three site data using a leave-one-out cross-validation. **A.** To avoid overfitting, we used an ensemble learning approach using the LS-boosting with a learning rate of 0.05 and the number of learning cycles of 100, leading to a convergence after 20 cycles. **B.** The inclusion of the site variable in the RFR and performing the ensemble method resulted in the mean MAE of 9.7 and r = 0.90 across the three site datasets, which was similar to the result when the post-hoc correction was used (left).
